# Cellular activation pathways and interaction networks in vascularized composite allotransplantation

**DOI:** 10.3389/fimmu.2023.1179355

**Published:** 2023-05-17

**Authors:** Leonard Knoedler, Samuel Knoedler, Adriana C. Panayi, Catherine A. A. Lee, Sam Sadigh, Lioba Huelsboemer, Viola A. Stoegner, Andreas Schroeter, Barbara Kern, Vikram Mookerjee, Christine G. Lian, Stefan G. Tullius, George F. Murphy, Bohdan Pomahac, Martin Kauke-Navarro

**Affiliations:** ^1^ Department of Plastic, Hand and Reconstructive Surgery, University Hospital Regensburg, Regensburg, Germany; ^2^ Division of Plastic Surgery, Department of Surgery, Yale New Haven Hospital, Yale School of Medicine, New Haven, CT, United States; ^3^ Department of Surgery, Division of Plastic Surgery, Brigham and Women’s Hospital, Harvard Medical School, Boston, MA, United States; ^4^ Department of Hand, Plastic and Reconstructive Surgery, Microsurgery, Burn Center, BG Trauma Center Ludwigshafen, University of Heidelberg, Ludwigshafen, Germany; ^5^ Department of Pathology, Brigham and Women’s Hospital, Boston, MA, United States; ^6^ Department of Plastic, Aesthetic, Hand and Reconstructive Surgery, Burn Center, Hannover Medical School, Hannover, Germany; ^7^ Division of Transplant Surgery, Department of Surgery, Brigham and Women’s Hospital, Harvard Medical School, Boston, MA, United States; ^8^ Department of Plastic Surgery, Charité – Universitätsmedizin Berlin, Corporate Member of Freie Universität Berlin, Humboldt-Universität zu Berlin and Berlin Institute of Health, Berlin, Germany

**Keywords:** transplant, reconstructive surgery, vascularized composite allotransplantation, VCA, alloimmune response, acute rejection, chronic rejection

## Abstract

Vascularized composite allotransplantation (VCA) is an evolving field of reconstructive surgery that has revolutionized the treatment of patients with devastating injuries, including those with limb losses or facial disfigurement. The transplanted units are typically comprised of different tissue types, including skin, mucosa, blood and lymphatic vasculature, muscle, and bone. It is widely accepted that the antigenicity of some VCA components, such as skin, is particularly potent in eliciting a strong recipient rejection response following transplantation. The fine line between tolerance and rejection of the graft is orchestrated by different cell types, including both donor and recipient-derived lymphocytes, macrophages, and other immune and donor-derived tissue cells (e.g., endothelium). Here, we delineate the role of different cell and tissue types during VCA rejection. Rejection of VCA grafts and the necessity of life-long multidrug immunosuppression remains one of the major challenges in this field. This review sheds light on recent developments in decoding the cellular signature of graft rejection in VCA and how these may, ultimately, influence the clinical management of VCA patients by way of novel therapies that target specific cellular processes.

## 1 Introduction

Devastating injuries such as severe facial disfigurement (e.g., loss of nose and lips) that cannot be adequately addressed by conventional reconstructive surgical techniques, including local tissue rearrangement and free tissue transfer. Although flaps, grafts, or local tissue rearrangement are the gold standard for reconstruction, they merely achieve wound coverage but result in substantial amount of donor-site morbidity. Vascularized composite allotransplants (e.g., face or limb transplants) have revolutionized functional and aesthetic restoration with promising short and long-term outcomes (1–3). Yet, this groundbreaking reconstructive biotechnology is also associated with challenges, mainly owing to the strong rejection response that invariably occurs following transplantation. VCAs are composed of a variety of tissue types, including, but not limited to, skin, mucosa, blood vessels, lymphatics, nerves, muscle, and bone, with suspected varying degrees of antigenicity between the tissues (4). To ensure graft survival, patients are usually maintained on high doses of systemic immunosuppression with potentially severe long-term side effects (5).

Different cell types such as endothelial cells (EC), B cells, T cell variants, natural killer (NK) cells, and antigen-presenting cells (APC) have been implicated in the pathogenesis of rejection. Based on the current evidence, there is a clear hierarchy of effector-target cell interactions. For acute cellular rejection, CD8^+^ T cells of both donor and recipient origin are the primary effector cells targeting epithelial and follicular stem cells and microvascular endothelium. During chronic rejection, the most relevant effector-target cell combinations are donor and recipient CD8^+^ effector T cells that target endothelium (partially chimeric), leading to arteritis with chronic remodeling. Of note, CD4^+^ T cells and antibodies seem to play an additional pivotal role in this process (6, 7). Secondary effector and modulator cells of the immune response (NK-cells, endothelial cells, granulocytes, T_regs_, macrophages and APC, immunosuppressive dermal mesenchymal cells, keratinocytes, mast cells) have also been recognized to play a role in modulating the primary effector-target cell interactions (8–11). Additionally, it has been demonstrated that both donor and recipient-derived immune cells may contribute to the development of allograft rejection (12). Given the recognition of CD8^+^ effector T cells as the main (but not only) protagonists of VCA allograft rejection, this cell type is predominantly targeted by current clinical immunosuppression regimens (Tacrolimus, Steroids, mycophenolate mofetil (MMF)) (13). To further improve our understanding of VCA pathoimmunology, there is a critical need to review the current understanding of how different cell types contribute to VCA rejection (**Supplementary Figure 1**).

## 2 Rejection in VCA – what tissue is the primary target?

VCA grafts are comprised of different tissue types (e.g., muscle, skin, mucosa, lymphatics, vasculature, adipose tissue), each of which may feature specific immunological properties and thus be differentially targeted by rejection (5, 14). Skin is largely viewed as the most immunogenic tissue and most studies focus on the rejection mechanisms in skin itself. The phenomenon of split tolerance (rejection of one tissue with tolerance towards another) has been observed in several studies, characterized by the rejection of skin components without evidence of rejection in the remaining tissue types (13, 15–17). The preferential targeting of the donor skin by the recipient immune system might be due to the transfer of a rich skin-resident donor-derived immune system, including T cells and APC (such as dendritic cells). It has been shown that the skin is home to more resident T cells when compared to the peripheral circulation, and given the constant exposure of skin to foreign antigens, a large number of T effector cells are present in the skin (particularly CD8^+^ memory T cells) (17). These cells potentially fuel detrimental interactions between the two immune (i.e., innate and adaptive) systems (12). Recent work by our group identified the mucosal tissue of the oral and nasal cavities as another main target of rejection in facial VCAs (14, 15, 18, 19). Interestingly, the mucosa consistently shows more distinct microscopic changes indicative of acute rejection events when compared to skin biopsies (14, 15, 20). Since most studies on comparative antigenicity were performed in limb allografts, the dogma that skin is the most immunogenic may need to be re-evaluated.

## 3 The hierarchy of effector-target cell interactions: cells and their relevance in VCA rejection

Few studies have detailed the precise role of different cell types involved in allograft rejection. However, since most therapeutics target a specific cell type (predominantly T and/or B cells), it is important to examine the relevance of specific cell types for allograft rejection and how these cell types interact. To decipher the hierarchy of effector-target cell interactions in allograft rejection, we will first delineate the functions of the different cell types in acute rejection. Of note, the transition from acute rejection episodes into chronic stages represents most likely a gradual process rather than two clearly differentiable phases (21, 22). Thus, the cell types discussed in this review are likely to be involved in both acute and chronic rejection. Yet, further research, especially on chronic VCA rejection, is needed to match cell type and fate with either acute and/or chronic rejection.

Mounting body of evidence points toward CD8^+^ effector T cells as the hallmark effectors of VCA rejection targeting epidermal and follicular stem cells (and to a lesser extent the microvascular endothelium), thus driving acute cellular rejection reactions (16, 23, 24). Besides these key effector-target cell interactions, multiple secondary effectors, in addition to other cellular targets, are involved in VCA rejection (17).

In acute VCA rejection, the primary effector-target cell pair are CD8^+^-T-cells and epithelial/follicular stem cells (25–28). This interaction will be discussed first followed by an outline of the role of endothelial cells which represent both targets and effectors in VCA. Lastly, secondary effectors (e.g., NK cells, APC, granulocytes with APC-like cellular characteristics) will be discussed (11, 29–31).

## 4 Acute allograft rejection

Acute rejection (AR) is usually defined as a typically reversible immune-mediated attack on the allograft, orchestrated at the cellular level by cytotoxic cells (predominantly T cells) or through donor-specific antibodies (DSA) produced by plasma cells (antibody mediated rejection, AMR). The primary effector/target cell combination are T cells that target epidermal and follicular stem cells. Nearly 85% of VCA patients experience at least one episode of AR within the first postoperative year; in fact, more than 50% of cases report multiple AR episodes (32). The reported incidence of AR is, thus, significantly higher than in the field of solid organ transplantation (SOT) (33). It is hypothesized that this is, at least partly, due to easy visual monitoring of the graft (8). Indeed, any suspicious skin changes or cutaneous abnormality should trigger a prompt biopsy and histologic examination. In cases of histologically confirmed AR, immediate therapy is often initiated, usually in the form of steroid bolus administration and immunotherapeutic regimen optimization (34). Of note, Win et al. found distinctive genetic features in AMR (on postoperative day 5) compared with T cell mediated rejection episodes (by 12, 21, and 24 months postoperative) in one VCA facial allograft. Genes that were particularly upregulated during the suspected AMR episode included ICAM1, VCAM1, and SELE, which are all linked with endothelial activation and leukocyte-endothelial cell interactions. In contrast, granzyme B was specifically upregulated during the T cell mediated rejection episodes. Granzyme B is commonly expressed by CD8^+^ cytotoxic T cells and natural killer cells upon activation (35).

**Figure 1:**
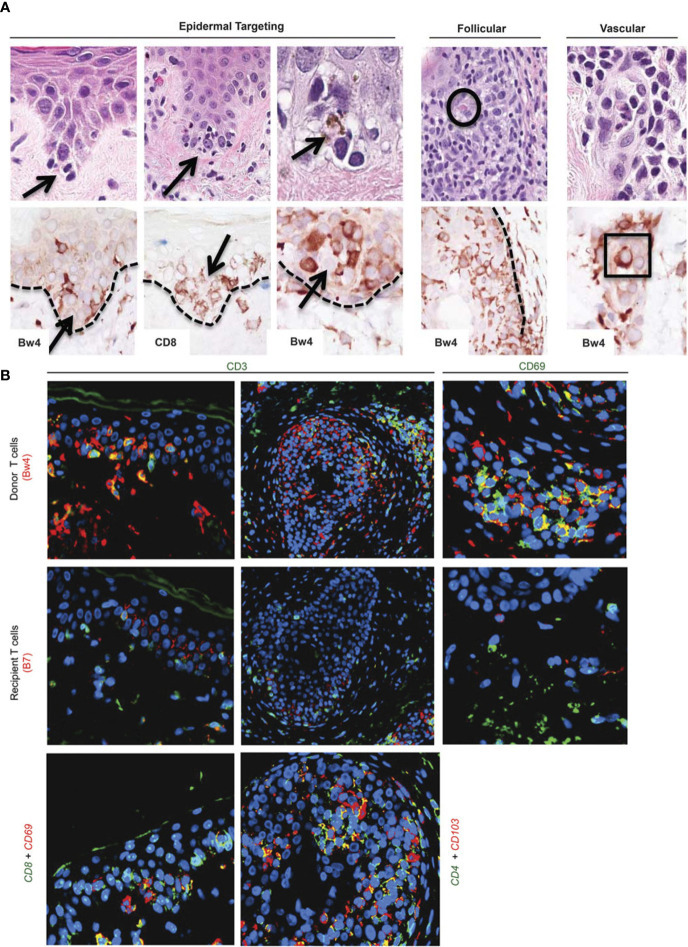
Target cells in acute rejection of facial VCA skin. **(A)** Spatial associations of lymphoid cells with target cell injury in facial allografts. Lymphocytes at tips of epidermal rete ridges surrounded target cells (arrows, top row H&E staining) to form ‘satellitosis’. Donor (Bw4) and CD8^+^ T cells precisely corresponded to these patterns of satellitosis (bottom row). Sites of follicular targeting in the bulge region, as well as microvascular targeting, also correlated with the presence of donor T cells that often were located within vessel lumens (intraluminal donor T cell in apposition to degenerating endothelial cells within square; intrafollicular apoptotic target cell encircled). Dotted line=dermal–epidermal and dermal–follicular junctions). **(B)** Dual labeling for donor/recipient histocompatibility antigens and T-cell phenotype (color of font=fluorochrome used; patient 5). The majority of cells in epidermal infiltrates (top and middle rows, far left) and follicular infiltrates (top and middle rows, middle) were CD3-positive (green) T cells of donor origin (Bw4, red; co-expression=yellow–orange). Cells expressing resident memory T-cell markers (CD69, green) co-expressed donor (Bw4, red) but not recipient (B7) antigen markers (top and middle rows, far right) consistent with their origin in the facial allograft. Donor resident memory T cells (CD69- or CD103-positive cells, red) were predominantly CD8 positive (green) in the epidermis and CD4 positive (green) in hair follicles (bottom row). Color mixing of red and green epitopes=yellow or yellow–orange; blue=DAPI nuclear stain. Please note: Figure 1 and the respective caption are adapted and modified from Lian et al. (12). Permission to reuse was granted by Springer Nature.

Histologically, AR of the skin in VCA shows different levels of perivascular and/or interstitial mononuclear cell infiltration with epidermal and/or adnexal involvement (36) (**Figure 1**). Bhan et al. provided initial evidence in 1980 that both CD4^+^ and CD8^+^ T-cells infiltrate human skin allografts (37). In a study of 113 biopsies from full-facial transplants, Lian et al. showed that T cells also play a crucial role during acute rejection of facial VCAs (12). Notably, Lian et al. found that some of the involved immune cells were of donor origin, with an immunophenotype typical of the resident memory T cell subset. While cell-mediated rejection primarily affects the skin in the sense of epidermal targeting, the endothelium of graft vessels is the main site of damage in antibody-mediated rejection (AMR) (36). During AMR, DSA trigger rejection by complement activation. In the classical pathway, along with C4a and C4b, C4d is subsequently cleaved and can covalently bind to the endothelium, serving as a diagnostic tool of AMR in solid organ transplantation (38, 39). Yet, AMR remains rarely reported in VCA recipients (40). In the few cases of AMR in VCA, no clear correlation between C4d deposition and DSA formation was found (41, 42).

## 5 T cells as the primary effector cells

### 5.1 Basic cellular characteristics of T-cells

T cells are the protagonists of adaptive cell-mediated immunity and the major cell type involved in VCA rejection (43). Two main populations of T-effector cells exist, which are divided based on their CD4 or CD8 glycoprotein that is associated with a respective TCR on the cell surface: CD4^+^ T-helper cells (T_H_-Cells) and CD8^+^ cytotoxic (cytolytic) T lymphocytes (CTL). T-helper cells produce cytokines and modulate immunological processes such as CTL, macrophage, and B-cell activation, whereas CTL bind to target cells and induce apoptosis in target cells *via* secretion of perforins and granzyme. These cell populations were noted to be involved in skin allograft rejection as early as in 1982 by Bhan et al. At the time, two pathways of T cell-mediated rejection mediated by T cells of recipient origin were proposed: a direct pathway of epithelial injury mediated by CD8^+^ CTLs, and an indirect pathway *via* T cell-mediated endothelial microvascular injury (37). With the clinical advent of VCA, we were able to better characterize functions and cell populations involved in skin rejection.

### 5.2 T cells as cornerstones of transplant immunity

T cells are specifically trained to recognize donor-derived antigens. The indirect pathway of antigen presentation is the conventional mechanism of antigen presentation of bacterial/viral antigens, whereas dendritic cells acquire an antigen *via* endocytosis, process it into peptide fragments, and present it on their surface *via* MHC molecules (= antigen presentation) (8, 16, 43). A processed antigen fragment (peptide) is presented on the surface of APC *via* self MHC I (for CD8^+^) or II (for CD4^+^ T cells), and the T cell binds *via* TCR. During transplant rejection, processed antigen is mostly a donor-derived MHC molecule. In the direct pathway of antigen recognition, T cells recognize intact (unprocessed) foreign MHC molecules on the surface of donor-derived cells (44). Naïve T cell activation requires professional APC (dendritic cell) activation to become an effector T cell. In addition to the antigen-specific signal, a costimulatory signal is required for T cell activation (43, 44). The B7:CD28 and CD80/CD86:CD40 pathways are the most widely known costimulatory pathways, and the costimulatory molecules are expressed in high density on APC.

### 5.3 T cells in VCA grafts and rejection

Cellular infiltrates of human skin rejection samples in hand transplantation consist predominantly of CD4+ and CD8+ lymphocytes, with some B-cells and CD68+ cells (histiocytes, macrophages) (45). In a detailed analysis of facial VCA skin samples, Lian et al. demonstrated that stem cell-rich epidermal rete ridges, follicles, and dermal microvessels were primarily targeted during the acute rejection process (12). Initially, it was assumed that most of the target cell damage is caused by recipient-derived lymphocytes that infiltrate the graft and react towards alloantigens. However, Lian et al. identified that both donor- and recipient-derived T-lymphocytes are involved in the rejection process. In the epidermis and hair follicles, most infiltrating lymphocytes were of donor origin (>90%) and stained positive mainly for CD8 (8). The authors demonstrated that lymphocytes surround epithelial target cells (“satellitosis”). Near vasculature, both donor and recipient lymphocytes of comparable quantity were identified, with donor-derived T cells often located intraluminally next to the injured endothelium (lymphocytic vasculitis). Multiplex gene expression profiling of both facial and limb VCA samples has recently been performed (13, 46). In face transplant samples, findings demonstrated that in higher grade rejection, mostly T cell activation pathways are activated with upregulation of genes associated with T cell infiltration (CD3D, CD3G, CD4, CD8A), T cell co-stimulation (TNFRSF4, CD28, ICOS, TNFRSF9), Th1 chemokines (e.g. CXCL9,10,11) and effector molecules such as granzyme A, granzyme B, granzyme K that are responsible for cytotoxicity (13). In biopsy samples from three limb transplant recipients, chemokines including CCL18 were shown to be significantly upregulated during allograft rejection. CCL18, which commonly binds to the CCR8 receptor, has been linked to an increased recruitment of allo-T cells (CD4^+^ and CD8^+^ T cells) to skin xenografts, leading to signs of accelerated graft rejection. Using a humanized skin transplantation model, the authors could show that blockade of CCR8 remarkedly decreased CCL18-induced allo-T cell infiltration (46).

## 6 Epithelial and follicular stem cells as primary target cells

Stem cells represent unspecialized cells of the human body (47). Following several steps of specialization, developmental potency is reduced with each step (48). Of note, both epithelial and follicular stem cells are characterized by high developmental capacity, which classifies them as multipotent stem cells. Epithelial stem cells, for example, can differentiate into keratinocytes (49). Follicular stem cells, in turn, have shown even stronger multipotency and can differentiate into various cell types such as keratinocytes, melanocytes, mesenchymal cells, neurons, and glial cells. In addition, they have also been shown to contribute to angiogenesis (50, 51). Stem cells are also involved in various immune-related diseases, such as cancer, cardiovascular, and autoimmune diseases, where they can both promote disease and contribute to healing (52–54). Cancer stem cells, for example, have been implicated with tumorigenesis and therapy resistance while other stem cell types can promote tissue regeneration and healing in cardiovascular and autoimmune diseases (55, 56). In acute VCA rejection, epithelial and follicular stem cells have been proposed as the primary target cells (12).

Cutaneous stem cells, such as epithelial and follicular stem cells, play an important role in skin regeneration, and complex interactions with skin resident and infiltrating immune cells have been observed. These interactions not only make the skin particularly susceptible for immune-related diseases like autoimmune disorders (e.g., systemic lupus erythematosus) but also may play a pivotal role in allograft rejection. Epithelial stem cells, for example, can be affected by various immune cell types including T_regs_ and macrophages. T_regs_ in close spatial proximity to hair follicles have been demonstrated to regulate the activity of follicular stem cells, thus impacting hair growth (57). Macrophages, in turn, have been shown to exert suppressive effects on follicular stem cells (58). Since hair follicles have the capacity to recruit a variety of immune cells in response to damage, follicular stem cells may be at particular risk of immune cell targeting and subsequent inflammation (59). The above-mentioned interactions may be based on various specific properties of cutaneous stem cells. For instance, several signaling pathways including JAK-STAT, β-catenin/Wnt, and Jag1-Notch exist, mediating the interaction of cutaneous stem cells with immune cells (60). Of additional interest, transcriptional profiling of human epithelial stem cells has shown increased expression of encoding surface receptors and cell adhesion molecules such as leukocyte differentiation antigens and activated leukocyte cell adhesion molecule (ALCAM), potentially further enhancing interactions with immune cells (61). Most interestingly, it has been shown that MHC class I and II expression can be increased in follicular stem cells under pathological conditions such as autoimmune disease, thus enhancing the potential for immune cell interactions. Intriguingly, this mechanism may also play a role in alloimmunity and explain why the skin is seen as a predominant target of the immune response in VCA (62, 63). In line with this observation, biopsies from facial transplant recipients, for example, have shown lymphocyte accumulation in epithelial stem cell-rich regions. Of note, intra-epidermal and intra-follicular cells were mainly targeted by cells exhibiting a donor-derived T cell phenotype (12). This was observed in a manner that mirrors other cytotoxic immune reactions in the skin such as acute graft-*versus*-host disease. Additional studies in face transplant recipients have reported that hair follicles are also involved in chronic rejection with hair loss as an early feature presenting prior to sclerosis or vasculopathy (64). More importantly, our own group has described epidermal thinning with a loss of rete ridges in chronic face transplant rejection (65). As epidermal stem cells are located within rete ridges, this indicates that skin stem cells do not only represent a target of alloimmunity in acute, but also in chronic rejection (66). In addition, studies of cutaneous graft versus host disease after bone marrow transplant have shown that epithelial stem cells are particularly targeted by effector T cells. Mechanistically, they have been shown to be especially prone to the pro-apoptotic effects of cytotoxic cytokines produced by allostimulated T cells (67). Of note, this susceptibility may be due to increased expression of cytokine-inducible adhesion molecules such as CD106 (68). Moreover, it has been shown that proinflammatory cytokines such as TNF-α may prime epithelial stem cells towards apoptosis by interacting with apoptosis-regulators such as p73, a member of the p53 family. This may ultimately result in increased vulnerability to the effects of allostimulated T cells (69). Taken together, these mechanisms may also play an important role in VCA rejection, thus potentially explaining the predominant targeting of skin stem cells.

## 7 Primary target and effector cells - endothelial cells

### 7.1 General cellular characteristics of endothelial cells

EC are the first line of defense against blood-borne pathogens and represent the interface between graft tissue and recipient-derived immune cells following VCA (70). In general, EC can act as “semiprofessional APC”, meaning that they can express the genes involved in antigen processing and presentation while lacking the surface receptors CD80 and CD86 (71). Furthermore, EC drive regeneration of the vasculature (72) and they can promote a local antithrombotic environment by activation of antithrombin-3 (73). Platt et al. have demonstrated that the activated complement system (i.e., the membrane attack complex (MAC)) and preformed DSAs trigger intravascular coagulation and EC vulnerability to oxygen radicals (74). In response to these processes, EC acquire pro-coagulant functions followed by activation of the tissue factor (TF) and thrombus formation (75–77).

### 7.2 Endothelial cells in VCA rejection

EC hold a key position in acute VCA rejection as they activate and recruit lymphocytes through the upregulated expression of HLA-II and adhesion molecules (e.g., E-selectin) on their cell surface (12). Ligation to the HLA-II molecule drives phosphorylation of protein kinase B (PKB), which increases IL-6 secretion of EC (78). Rising IL-6 levels contribute to the expansion of the T_h_17 subset of T helper cells while reducing the immunosuppressive regulatory T cells (T_regs_). The T_h_17 subset has been shown to navigate neutrophil granulocytes to the inflammation site *via* IL-8 and produce proinflammatory IL-17A and IL-22 (79). Furthermore, E-selectin orchestrates overall leukocyte trafficking and allows transendothelial migration of lymphocytes to the skin. This migration process is due to T cells harboring the cutaneous lymphocyte antigen (CLA; a ligand for E-selectin) which is commonly expressed on <5% of T cells. However, in skin lesion biopsies, >80% of T cells were found to be CLA^+^ (80). Further, EC can lead to increased levels of vasoactive messenger substances such as bradykinin, prostacyclin, and nitric oxide (NO) during acute VCA rejection (12, 81). This can be due to increased levels of IL-6 resulting in elevated NO levels (82). Mechanistically, high-level NO leads to phosphorylation of vascular endothelial (VE)-cadherin Y685 which promotes the dismantling of adherens junctions between the EC (83). This cascade entails the disruption of the endothelial barrier that is crucial for the fluid, gas, and metabolic homeostasis of the VCA transplant (84). Because of the limited vessel diameters, especially at the capillary level (≤5 μm versus activated T cells measuring 8- 12 μm), the EC inevitably interact with T cells (85). This phenomenon may explain how EC can activate resting memory T cells in acute VCA rejection, unlike for example fibroblasts, despite both cell types expressing HLA-II molecules (85).

During acute VCA rejection, EC can be involved in cellular- (CMR) or antibody-mediated rejection (AMR) processes. In terms of CMR, EC of the allograft act *via* the presentation of donor HLA molecules which are recognized by T cells and trigger inflammatory pathways. Further, VCA grafts host an array of expanded populations of donor T cells expressing CD69, CD103, and CLA (i.e., markers of resident memory T cells (T_rm_)). The increased prevalence of this cell subset can be due to the majority of VCA patients having undergone previous blood transfusions and/or surgical procedures, thereby inducing donor antigen sensitization and generating long-lived (i.e., occurring up to 23 months following facial transplants), donor-specific memory T cells (12, 86). This subset has been implicated with early-onset VCA rejection on postoperative day five. Vice versa, it remains to be determined whether donor-derived T cells target recipient EC that are derived from blood vessels that grow into the VCA graft (12). Remarkably, AMR has thus far occurred less frequently in VCA than in SOT. This might be due to the ingrowth of recipient blood vessels and/or migration of EC into the VCA graft, which prevents binding of DSA to the EC (25). Further, tissue damage in the aftermath of VCA surgery may trigger the release of donor antigens which can lead to clonal suppression (i.e., they interact with the respective B cell antigen receptors and suppress the production of DSA) as well as directly bind to DSA, hindering their interaction with donor EC (87). The deposition of C4d on EC – a hallmark of AMR in SOT – has also been demonstrated in bilateral hand transplant recipients, as well as forearm and full-face transplants, but its diagnostic value in VCA rejection remains controversial (10, 41).

EC lining the endothelial wall in deep donor arteries, as well as synovial and sentinel skin graft vessels, have been associated with chronic VCA graft rejection (88). The first face transplant patient re-presented with fibrosing EC, which impaired the functionality of the endothelial barrier and (presumably) caused EC-driven thrombosis of VCA vessels (88). The Lyon group outlined the key role of capillary thrombosis in chronic VCA rejection after studying 10 VCA recipients (21). Interestingly, the involvement of fibrosing EC in chronic VCA rejection has also been proposed by Mundinger et al. utilizing a nonhuman primate model of face transplantation (89). Lymphoid follicle formation, which has been reported in chronic VCA rejection by Mundinger et al. but also separately in hand transplants, seems to be intertwined with EC: follicles were found to be surrounded by endothelial venule-like vessels (89, 90). In addition, accelerated arteriosclerosis has been found in SOT, which may be mediated by various processes including ischemic injury of vessels during transplant, viral infections of the graft, and side effects of immunosuppressive drugs such as corticosteroid-induced hyperlipoproteinemia (91). Of note, arteriosclerosis-like changes such as intimal thickening have also been observed in acute VCA rejection. Moreover, rejecting VCA patients are often treated with high-dose corticosteroids. Thus, accelerated arteriosclerosis may play a key role in chronic VCA rejection and contribute to graft damage (92).

### 7.3 Targeted therapeutic strategies

In terms of medication regimens targeting EC in VCA rejection, ustekinumab and secukinumab have demonstrated beneficial effects by inhibiting T_h_17 cell proliferation and blocking IL-17A in SOT and were therefore tested in an osteomyocutaneous radial forearm flap model of non-human primates by Atia et al. (93, 94). Of note, the authors combined ustekinumab and secukinumab with belatacept, which blocks CD86-CD28 interactions, but they did not find improved outcomes when compared to the standard triple immunosuppressive regimen (i.e., tacrolimus, mycophenolate mofetil (MMF), and methylprednisolone). They hypothesized that those results were based on CD28-independent co-signaling pathways in T_h_17 cells and high-level CTLA-4 expression inducing resistance to belatacept (95). Complement system activation leading to C4d disposition on endothelia as the hallmark of AMR represents another drug target in VCA medication therapy. To this end, eculizumab that blocks the cleavage of C5 into C5a and C5b fragments has been applied in a highly presensitized full-face transplant patient in combination with total plasma exchange therapy and intravenous immunoglobulin administration. This drug protocol antagonized symptoms of VCA rejection in a full-face allotransplant recipient within 1-month post-transplantation and allowed reduced medication therapy compared to the standard triple immunosuppression regiment (41). Furthermore, when investigating the molecular biology of skin rejection in VCA, Hautz et al. observed a close correlation between the severity of rejection with ICAM-1 and E-selectin (45). Both E- and P-selection mediate the binding and rolling of immune cells in the vasculature (96, 97). The therapeutic potential of these adhesion molecules was then investigated in a rat hind limb allotransplant model by locally administering efomycine E (a specific E- and P-selectin inhibitor) (45). When combined with antithymocyte globulin and low-dose tacrolimus, a weekly subcutaneous injection of efomycine E led to a significantly prolonged skin and allograft survival. Thus, targeting adhesion molecules may be a promising adjunctive treatment to reduce the burdensome immunosuppressive regimen. Nevertheless, the administration of efomycine E alone remained ineffective, indicating the need for combined therapies.

## 8 Secondary effector cells – from natural killer cells to mast cells

### 8.1 Natural killer cells – uncharted secondary effectors?

#### 8.1.1 The interplay of natural killer cells and the immune system

NK cells are effector lymphocytes of the innate immune system. While NK cells lack the clonotypic TCR and CD3ϵ (i.e., the associated signal-transducing adaptor), they express the low-affinity Fc receptor CD16 and are therefore capable of detecting antibody-coated target cells, exerting antibody-dependent cell cytotoxicity. NK cell activation is initiated by the interplay of different activating and inhibitory signals involving ITAM (immunoreceptor tyrosine-based activation motif)-bearing molecules (98). NK cells can secrete the cytotoxic reagents perforin and granzyme B (99). Further, NK cells interact with EC for example *via* binding of α4β1 integrin to vascular cell adhesion protein-1 (VCAM-1) (100). NK cells can also mediate DC homeostasis *via* IFN-γ and TNF-α. NK cells can prime T_h_1 cells through IFN-γ secretion (101). Regarding B cells, NK cells have been demonstrated to increase IgG and IgM antibody production and facilitate immunoglobulin class switching (102, 103).

#### 8.1.2 Natural killer cells in VCA rejection reaction

Friedman et al. identified multiple cytokines (e.g., IL-10 and IL-18) and chemokines (e.g., CXCL-9 and CX3CL-1) that were upregulated in VCA rejection (104). CX3CL-1 is expressed by EC and directs C-X3-C motif receptor-1 (CX3CR-1) expressing cells like macrophages and NK cells into inflammatory sites. In response to this ligation, NK cells produce IFN-γ which drives CX3CL-1 expression on EC, pointing towards a paracrine feedback loop between CX3CL-1 and CX3CR-1 (105). IFN-γ induces T_h_1 responses and increases ROS levels, leading to endothelial damage in VCA grafts (104). Moris et al. further proposed an activating effect of T_rm_ on NK cells in VCA rejection (24). In NK cells, this interaction coordinates increased granzyme B secretion (106). Granzyme B, a serine protease found in the lytic granules of NK cells, orchestrates cell apoptosis, especially through Bid (i.e., a Bcl-2 family member) (107). Interestingly, elevated levels of granzyme B have been shown to increase mesenchymal stem cell (MSC) populations (108). Conversely, MSCs suppress the activity of NK and other immune cells for example by increasing tryptophan metabolites (109, 110). Kuo et al. translated the basic biological links between NK cells and MSCs into the field of VCA and corroborated the modulatory effects of MSCs on NK cells (109). Of note, Win et al. directly compared the potency of NK cells versus CD3^+^ T cells during rejection by collecting skin biopsies from 7 face transplant patients. The authors revealed that T cells accounted for the majority of secreted granzyme B and outnumbered NK cells 4-fold. Yet, their work demonstrated that 56% of NK cells were activated (i.e., CD56^+^CD107^+^) in comparison to 21% of CD3^+^ T cells expressing the activation marker CD40L. To evaluate the relative contributions of T cells and NK cells to cytotoxic injury, the authors immunostained for caspase-8 (a marker of cytotoxic cell death) and found that T cells mediated significantly more cytotoxicity than NK cells and were responsible for a mean 71% of cytotoxic events in VCA grafts (versus 29% attributed to NK cell cytotoxicity) (13). While the relative NK cell count following VCA surgery remains to be ascertained, preliminary data from Belike et al. demonstrated that anti-NK treatment (HB-191) administered one day prior to transplantation of islet allografts did not delay acute rejection in a diabetic murine model (111). Their findings may point towards a subordinate role of NK cells in the acute rejection phase which remains to be investigated in further studies.

#### 8.1.3 Pharmacological modulation of natural killer cells

NK cells in VCA rejection can be targeted by repetitive injections of MSCs. Their potent effects have been demonstrated in a rodent hindlimb model by Kuo et al. They found that the combination of adipose-derived MSCs (administered on day 7, 14, and 21 post-transplantation), anti-lymphocyte serum, and cyclosporin A yielded prolonged allograft survival when compared to the untreated control and the subgroup receiving only anti-lymphocyte serum and cyclosporin A (112). The same group confirmed the beneficial use of MSC injections for VCA graft survival in both a swine hindlimb model as well as a swine hemifacial model (113, 114). Expanding on the modulatory effects of MSCs on NK cells, there is a complex interplay between these cell types. MSCs can shield against NK cell-mediated lysis by expressing serine protease inhibitor 9 or TLR-3 (115). Vice versa, IDO and prostaglandin E2 (PGE2) are pivotal mediators in MSC-induced suppression of NK cells as they interfere with IL-2 which is crucial for NK cell proliferation (116). Friedman et al. introduced CX3CR-1 on the NK cell surface as a potential point of leverage in VCA rejection therapy (104). In murine models, CX3CR-1^-/-^ mice have shown prolonged cardiac allograft survival, though this remains to be translated to VCA settings (117).

## 9 Beyond antibody production - B cells as secondary effectors

### 9.1 Cellular characteristics of B cells

The bone marrow hosts the initial phases of B cell development and facilitates the deletion of B cells which bind self-antigens (i.e., negative selection) and the assembly of the B cell receptor. In response to antigen contact, naïve B cells differentiate into plasma or memory B cells (29, 118, 119). Memory B cell populations can shift into long-lived plasma cells or GC B cells following antigen rechallenge (29). The interplay with T follicular helper (T_fh_) cells further impacts B cell fate as T_fh_ cells act on GC B cells for example by IL-4 and IL-21 secretion and CD40L-CD40-interaction (120). B cell memory shields human health *via* long-lived plasma cells in the bone marrow that secrete highly specific antibodies recognizing recurrent homologous pathogens (121). Variant pathogens that may have evaded this first line of defense are targeted by memory B cells accumulating a broader set of antigen affinities and specificities (122). Mucosal B cells populate the gut, respiratory, and urogenital mucosae as well as the skin, salivary, mammary, and lacrimal glands. Their main purpose is to engage with secretory epithelia to provide a first-line defense through the secretion of immunoglobulin A (IgA) (123). B cells are also classified as APC as they form immunological synapses in an actin-dependent manner to capture, process, and present antigens on HLA-II molecules to CD4^+^ T cells upon B cell receptor signaling (124).

### 9.2 The crosstalk of B cells and VCA rejection process

The B cell-based production of DSAs holds a pivotal role in the overlap of B cell biology and VCA and can be subdivided into preexisting versus *de-novo* DSAs, with the latter typically occurring within 3 months post-transplantation (25, 40, 125, 126). In theory, the immunogenic components of VCA grafts (especially skin and mucosal tissue) and the frequently encountered presensitization (i.e., the formation of preformed potentially donor-specific antibodies) of VCA recipients through previous blood transfusions and/or surgical procedures may predispose this patient population to AMR. However, AMR is inconsistently observed in VCA patients. This might be due to (i) blood vessels in VCA grafts partially sprouting from the recipient’s vasculature and thus not expressing antigens recognized by DSA (this effect is potentiated by the fact that >80% of immunoglobulins circulate with the bloodstream, exposing the intravascular surface as the main location of arising alloreactivity), (ii) antigen shed from the graft resulting in clonal suppression (i.e., suppression of the DSA production) and enhancement (i.e., blockade of antigen recognition by DSAs), (iii) the interplay of DSAs with the graft tissue inducing resistance to AMR, and (iv) the prolonged normothermic ischemia time and higher frequency of reperfusions potentially reducing ischemic reperfusion injury (IRI) and development of DSAs (25, 42, 127). Further, the incidence of *de-novo* DSAs does not seem to be higher in VCA than SOT, as recent work by the Oxford VCA program found that 6/16 (37%) abdominal wall transplant patients developed *de-novo* DSAs versus eight of 13 (61%) intestinal or multi-visceral transplant recipients. Interestingly, they reported no case of AMR in their patient cohort, no difference in 1- and 3-year graft survival when stratified for the formation of *de-novo* DSAs, and they did not find any correlation between the incidence of *de-novo* DSAs and previous blood transfusions. The authors proposed that rejection in VCA patients was treated before triggering significant tissue damage and formation of *de-novo* DSAs (128). Following-up on six face transplant recipients, Borges et al. reported on one patient with AMR who – in contrast to the other recipients – displayed a persistent dominance of T_h_17 (129). This T cell subset has been implicated in the induction of B cell proliferation, increased antibody production cells, and elevated levels of the inducible T cell costimulator (ICOS) molecule further promoting B cell differentiation (130). Biopsies taken from the first face-allograft recipient nine years post-transplantation due to skin rejection symptoms showed dermal infiltration of CD20^+^ B cells in the facial allograft, as well as the sentinel skin graft. Here, CD20^+^ B cells formed tertiary lymphoid organs (TLOs), which are considered pathological hallmarks of chronic VCA rejection (88). Interestingly, TLOs TLS can mimic functions of GCs and drive the proliferation and/or differentiation of autoreactive B cells for example *via* the activation-induced cytidine deaminase (AID) (131).

### 9.3 B cell-focused pharmacological treatment protocols

Current immunosuppressive protocols are often focused on antagonizing alloreactive T cell responses with, for example, tacrolimus and cyclosporin A showing limited effects on B cells (40, 125). Sutter et al. utilized the Brown Norway-to-Lewis hind limb model to evaluate the effects of *in situ* forming rapamycin implants on VCA acceptance. While the authors observed increased levels of multilineage mixed chimerism and frequency of T_regs_, they found no significantly different B cell frequencies in peripheral blood and bone marrow when compared to the untreated control group (132). Belatacept is a fusion protein (CTLA4-IgG1) that targets the CD28/B7 co-stimulation between T and B cells resulting in inhibition of DSA formation, as shown by Grahammer et al. in four hand-transplanted patients (133). *De-novo* belatacept-based treatment has led to sufficient rejection prophylaxis and reduced side effects in a hand transplant recipient when compared to calcineurin inhibitor-based protocols (134). The Innsbruck group reported the first case of a primarily B cell-driven rejection episode in a forearm recipient at nine years post-transplant. While the patient did not respond to steroid treatment, administration of rituximab (i.e., an anti-CD20 monoclonal antibody) resulted in complete remission of clinical symptoms (10). While the therapeutic benefits of B_regs_ in controlling VCA rejection remain to be ascertained, the NK cell-mediated effects of B_regs_ in inducing allograft tolerance have been demonstrated by Schuetz et al. in a murine islet model. Of note, the authors did not find quantitative changes in B_regs_ population when NK cells had previously been depleted and therefore hypothesized that NK cells mainly influence the donor-specific regulatory function of B_regs_ (135).

## 10 APC and their role as secondary effector cells

### 10.1 Phenotypic and functional definition of antigen presenting cells

APC are involved in the rejection process *via* the direct and indirect pathways of allorecognition. In the direct pathway, donor-derived APC are recognized by recipient T-cells due to their foreign MHC molecules, whereas in the indirect pathway, recipient-derived APC have processed donor-derived antigens and present them *via* self-MHC to recipient T-cells (136). Dendritic cells (DCs), macrophages, and B cells represent professional APC and express pattern recognition receptors (PRRs) such as Toll-like receptors to recognize pathogen-associated molecular patterns (PAMPs), as well as damage-associated molecular patterns (DAMPs) (137). APC internalize the target molecule *via* endocytosis, pinocytosis, or phagocytosis (138). The internalized molecules are processed and presented by MHC-I to CD8^+^ T cells and MHC-II to CD4^+^ T helper cells, respectively. In a reciprocal manner, APC and T cells promote their interactions for example *via* co-stimulatory signal involving B7 molecules (i.e., CD80 and CD86) and CD28 or CD40 and CD40L (139–141).

### 10.2 Antigen presenting cells during VCA rejection

In facial VCAs, dendritic-appearing CD8^+^ T cells have been identified, representing a subclass of dendritic T_rm_ (12). DCs and CD8^+^ T_rm_ both express for example CD103L to bind E-cadherin, which is a hallmark in mucosal and epithelial barrier function (142). While they constitute a dendritic-*like*, not dendritic-*identical* phenotype, a potential cross-talk between DCs and T_rm_ in VCA seems plausible and has already been demonstrated in cancer research (143). Biopsy staining from four face transplants further revealed a novel role of tissue-resident macrophages in the surveillance of VCA graft integrity (144). Given their dependence on colony-stimulating factor 1 receptor (CSF1R), tissue-resident macrophages are defined as “M2-like” macrophages with a CD169^+^ subset displaying distinctive immunoregulatory properties (145). In keeping with results from rodent studies, Ualiyeva proposed the loss of this subset to be indicative of VCA rejection and identified T cell immunoglobin mucin-4 (TIM-4) as a potential driver of CD169^+^ tissue-resident macrophages migration to inflammatory/rejection sites *via* its phosphatidylserine binding pocket (144). Further, VCA graft-infiltrating macrophages produce IL-18 and reactive oxygen species (ROS) (104). IL-18 has been shown to trigger the secretion of proinflammatory interferon-γ (IFN-γ), and ROS can trigger pro-apoptotic B cell lymphoma-2 (Bcl-2) family proteins as well as impair mitochondrial function (146, 147) (for in-depth analysis of B cells in VCA please refer to “B cells”).

### 10.3 Pharmacological targets of antigen presenting cells

In VCA rejection, DCs can be therapeutically targeted by administration of the mammalian target of rapamycin (mTOR) inhibitor sirolimus (rapamycin) which conditions DC to stimulate immunosuppressive T_regs_ while reducing alloreactive T cell populations (148, 149). Interestingly, donor alloantigen-pulsed immature recipient DCs in combination with low-dose cyclosporine plus antilymphocyte serum regimen prolonged graft survival (32 versus 18 days in the cyclosporine-only control group) in a rat hindlimb model (150). This effect might be due to the induction of T cell hyporesponsiveness and reduced IFN-γ secretion. APC produce IL-12 leading to increased IL-4 levels, which have been demonstrated to promote M2 macrophage differentiation and function, as well as represent a marker for acute VCA rejection (151). This macrophage subset has been implicated with graft rejection (152, 153). Thus, targeting IL-4 *via* IL-4Rα blocker dupilumab seems an intriguing strategy for VCA rejection therapy and has already been successfully applied in the treatment of atopic dermatitis (154). Ustekinumab, an anti-IL-12/23 antibody, has shown potent effects in VCA rejection (please see “Endothelial Cells”) (93). Yet, its interplay with APC in the context of VCA remains to be determined.

## 11 APC-like cells – secondary effector cells in acute VCA rejection

### 11.1 Cellular cornerstones of granulocytes

Granulocytes are the most abundant subpopulation of leukocytes and can be subdivided into neutrophils, eosinophils, and basophils (155). Neutrophils express HLA-II and costimulatory molecules (e.g., CD80 and CD86) needed for antigen presentation and acquire DC-like functionality after exposure to cytokines (156, 157). Eosinophils have been involved shuttling of antigens to lymphoid tissues, while MHC-II molecules can be expressed in response to cytokine contact (158). Murine data points towards basophils acting as APC, while Eckl-Dorna et al. showed that basophils from allergic patients did not exert antigen-presenting functions (155, 159, 160).

### 11.2 The role of granulocytes in VCA rejection

Utilizing a groin free-flap rat model, Friedman et al. provided evidence of high overall granulocyte contents amongst infiltrating leukocytes within inflamed graft regions (104). The Innsbruck group reported that granulocytes were located interstitially and toward the epidermis in biopsies from a VCA patient eleven years after hand transplantation (161). Etra et al. found neutrophilic inflammation to be significantly correlated with severe VCA rejection in swine hindlimb transplants but not specific to the pathogenesis of rejection. They further identified granulocytes (neutrophils and eosinophils) in rejecting animal skin samples, whilst the vast majority of infiltrating inflammatory cells were lymphocytes (162). Overall, further studies are needed to determine the specific role of the different granulocytic subsets in VCA rejection and translate previous findings on the pharmacological targeting of granulocytes into the field of VCA.

### 11.3 Pharmacological modulation of granulocytes

Neutrophils express HLA-II and costimulatory molecules (e.g., CD80 and CD86) needed for antigen presentation with both immature and mature neutrophils being able to acquire DC-like functionality after exposure to cytokines such as IFN-γ, TNF-α, or IL-4. Alternatively, depletion of anti-granulocyte receptor-1 (Gr-1) or combined CXCR2–formyl peptide receptor 1 antagonism have been demonstrated to reduce neutrophil infiltration in a murine hepatocyte injury model (163). In allergy therapy, blocking antibodies (Benralizumab) targeting the IL-5 receptor, as well as anti-IL-5 neutralizing antibodies (Reslizumab) have been demonstrated to reduce eosinophils count (164, 165). Blocking key eosinophil molecules such as IL-33 and CD48 represents another therapeutic option. Addressing inhibitory receptors (e.g., Siglec-8 and CD300a) may allow eosinophils to be directly targeted, regardless of the underlying activating stimulus (166). CD48 and CD300a have also been researched in the context of basophil-centered allergy therapies (167). Mylotarg, a CD33-targeted drug, has been implicated with a reduced frequency of basophils without driving basophil-derived histamine secretion (168).

## 12 Additional cell types involved in acute VCA rejection

### 12.1 Secondary T-cell subset: regulatory T cells and their immunosuppressive effects

Regulatory T cells (i.e., CD4^+^CD25^+^CD127^-^ T cells; T_regs_) exert their immunosuppressive effects through direct cell-cell interaction with target immune cells or through the expression of immunosuppressive cytokines and anti-inflammatory molecules (169–171). Sagoo et al. demonstrated the beneficial effect of T_regs_ cells in preventing skin allograft rejection utilizing humanized mouse models (172). T_regs_ can be equipped with a chimeric antigen receptor (CAR) representing another, yet bioengineered cell type in VCA grafts (173). Such CAR-T_regs_ are not MHC-dependent in their activation process and instead specifically migrate to the rejection site, where they unleash more potent immunosuppression than conventional polyclonal T_regs_ (3).

### 12.2 Beyond epithelial and follicular stem cells

Different types of stem cells have been shown to play important immunoregulatory roles in allograft rejection, including MSC and adipose-derived stem cells (ASC). Experimental administration of MSC, for example, has been shown to mitigate acute allograft rejection in a hindlimb VCA model, thereby significantly prolonging graft survival. Mechanistically, these findings were associated with elevated levels of regulatory T cells (T_regs_) in the peripheral blood and skin of recipients. These observations are supported by *in vitro* evidence showing increased T_reg_ proliferation in co-cultures with MSC and T cells when compared to T cells alone. Of histological interest, MSC accumulated in the subcutaneous layer of both donor and recipient skin, whereas no significant accumulation in muscle or bone marrow tissue was detected (109, 174). Of note, another study investigating the effects of MSC in a tracheal transplant model also observed beneficial effects on allograft survival with augmented microvascular blood flow and oxygenation of the graft. Mechanistically, these results have been associated with upregulation of T_regs_ and increased levels of cytokines IL-5, IL-10, and IL-15 (175). ASC have also been reported to prolong allograft survival in experimental VCA models. Consistently, these observations have been associated with elevated T_reg_ levels in the peripheral blood and skin compartment of the allograft, along with increased TGF-β1 and IL-10 expression. Moreover, *in vitro* experiments demonstrated attenuating effects of ASC on T cell proliferation (112). Further studies have confirmed the regulatory effects of ASC on T cells with decreased T cell proliferation and increased numbers of T_regs_ (176). Of additional interest, ASC also appear to play an important role in regulating B cell responses in the setting of transplantation. More specifically, ASC administration was associated with increased amounts of CD45Ra^+^/Foxp3^+^ regulatory B cell subsets. Investigating complement activation in the same study, C4d, a marker of cellular- and antibody-mediated rejection, has been found to be decreased in transplanted alloskin tissue when administering ASC (177). Moreover, studies investigating the effects of ASC on graft vasculopathy have observed reduced intima/media ratios in arterioles of allograft skin and muscle, indicating beneficial effects of ASC treatment (178).

### 12.3 Langerhans cells – a special subtype of APC

Langerhans cells (LC) are a subset of immature dendritic cells and as such are a type of APC that can be found in the epidermis (179). Upon phagocytosis of antigens, they migrate to regional lymph nodes where they initiate T cell responses by antigen presentation (180). Most interestingly, LC have also been shown to exert immunosuppressive effects by selectively promoting the expansion and activation of skin-resident T_regs_ (181, 182). Hence, it can be assumed that LC not only possess stimulatory, but also regulatory capabilities, emphasizing the complexity of the skin immune microenvironment (181). In tolerated VCA, recipient-derived LC have been shown to rapidly infiltrate allografts after transplantation, thereby establishing LC chimerism. However, in rejecting VCA, initial dilution of graft-resident LC has been observed, followed by extensive infiltration by recipient-derived LC, ultimately leading to a near-complete loss of donor cell contribution. This infiltration of rejecting VCA most likely occurs *via* transvascular migration, as previous studies have observed perivascular infiltrates in early VCA rejection phases (16, 183, 184). In summary, these findings suggest that durable chimerism of LC is an important contributor to immune homeostasis and long-term graft tolerance in VCA (185). Moreover, previous studies have shown that large amounts of donor lymphocytes are released into the recipient circulation upon graft reperfusion, which may further stimulate recipient dendritic cells and thereby drive infiltration (186).

### 12.4 Mast cells – the key players in TNF-α-mediated VCA rejection?

Mast cells (tissue-resident, innate immune cells orchestrating inflammatory response and tissue homeostasis; MC) have also been shown to be implicated in allograft rejection. In experimental skin graft models, MC have been observed to promote allograft rejection by inducing proinflammatory responses through degranulation, with elevated levels of cytokines such as keratinocyte chemoattractant (KC), macrophage inflammatory protein 2 (MIP-2), and TNF-α. Consequently, increased infiltration of neutrophils has been described, further accelerating graft rejection. Accordingly, administration of MC-stabilizing drugs such as cromolyn significantly delayed allograft rejection (187). Of additional interest, studies investigating the role of mast cells in skeletal muscle ischemia reperfusion injury (IRI) observed increased muscle viability in mast cell-deficient mice following ischemic injury when compared to wild-type mice. Histologically, this was accompanied by increased muscle necrosis in wild-type animals, suggesting that MC are a major source of necrosis mediators in muscle IRI (188). Additional studies on VCA have shown that several inflammatory mediators, including IL-4, IL-12p70 and TNF-α, can serve as predictors for alloskin rejection. For muscle rejection, in turn, IL-12p70 and TNF-α have been identified as particularly accurate classifiers. Of note, MC appear to be the main source of TNF-α in the skin, entailing the initiation of T cell responses with subsequent tissue injury and allograft dysfunction. Thus, MC may be a key initiator of TNF-α-mediated rejection processes in VCA (189–191).

## 13 Chronic allograft rejection

Recently, several chronic rejection (CR) cases have been reported in VCA (17, 22, 92, 192, 193). Chronic rejection is typically defined as an irreversible later-onset change of the allograft that leads to loss of function and accelerated aging. The mechanisms leading to CR in fVCA are poorly understood but both cellular responses (against MHC bearing endothelium as proposed by Libby and Tanaka) and antibody mediated injury to graft vasculature may play a role (91). As sparse as the understanding is, the consequences of CR in VCA are drastic: CR in VCA irreversibly leads to gradual destruction, with graft dysfunction and loss of architecture as ultimate endpoints (88). In human VCA recipients, the skin and vasculature were identified as the main targets of CR (21, 22, 65, 192–196): Clinically documented manifestations of CR ranged from cutaneous lesions to changes in skin adnexa (loss of hair and nails). Krezdorn et al. collected biopsies of seven fVCA patients for up to an 8-year interval postoperative. The authors found that sclerotic zones in the allografts demonstrated upregulation of AP-1 pathway components such as JunB and c-Fos. These genes have been implicated with overproduction of type I dermal collagen in the setting of autoimmune cutaneous disorders (e.g., systemic sclerosis) (65). Further, Lee et al. used digital spatial proteomic profiling to show the expression of pathway components involved in atherogenic responses, including IDO1 and STING, as well as proteins expressed by activated cytotoxic T cells and macrophages (197).

**Figure 2:**
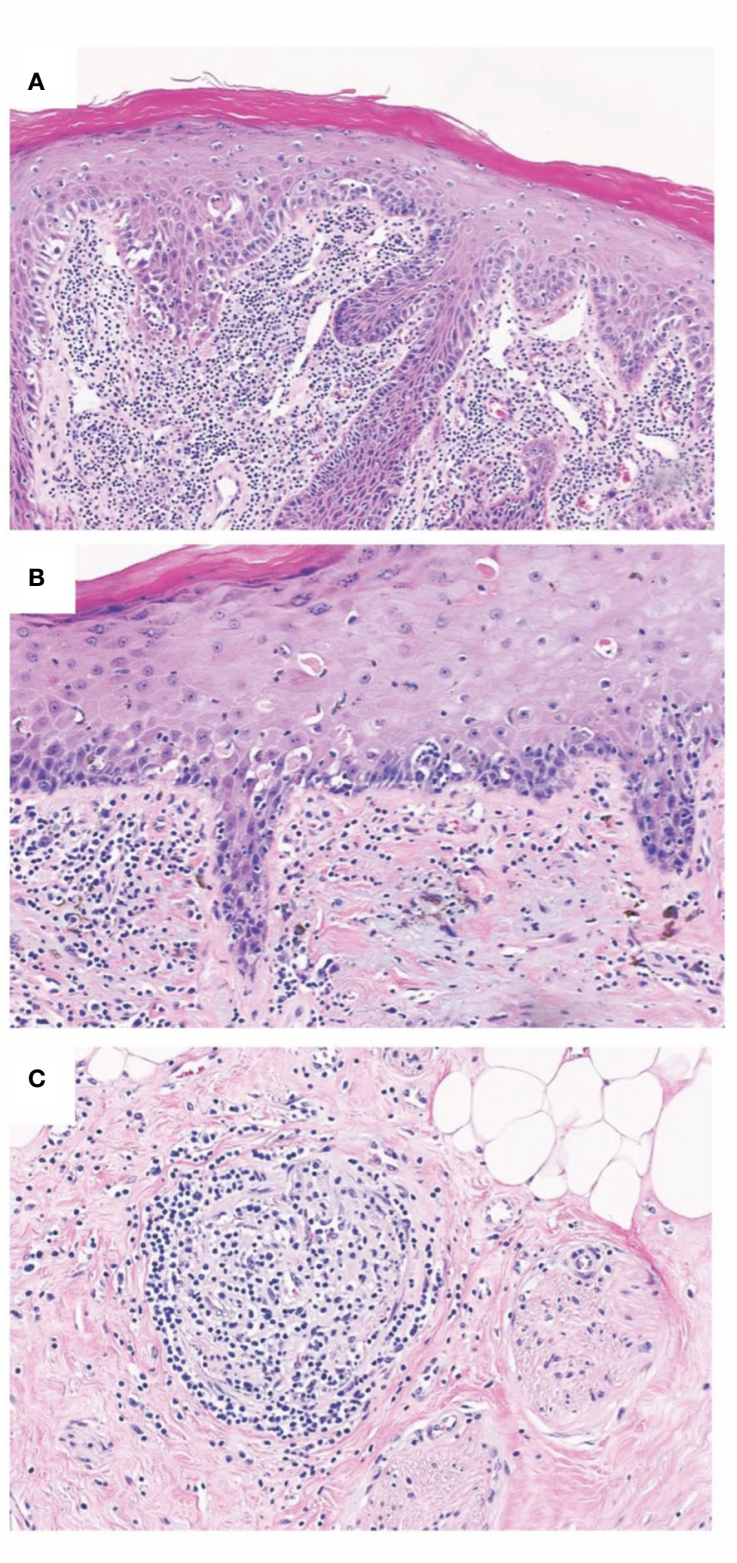
Chronic allograft changes in Fvca. Chronic rejection is associated with different histopathological changes such as allograft vasculopathy with capillary thrombosis, accelerated arteriosclerosis, as well as the destruction of the epidermal architecture. **(A)** Histopathological findings of T-cell mediated rejection involving lip mucosa of face transplant show interface change with scattered apoptotic target epithelial cells with associated dense subepithelial inflammatory infiltrate. **(B)** Chronic face transplant rejection of skin as shown in this biopsy specimen demonstrates epidermal thinning with intraepidermal colloid body formation and occasional lymphocyte-associated apoptotic keratinocytes. Evidence of chronicity in allograft transplant rejection also includes fibrous thickening of subepidermal basement membrane zone and deformation of hair follicles. **(C)** Representative image of lymphocytic vasculitis seen in acute and chronic rejections of VCA. Histopathology of deep dermal vascular injury shows lymphocytes surrounding and infiltrating vessel walls associated with endothelial cell damage.

The main pathological correlates of CR were chronic endothelial changes leading to graft vasculopathy with capillary thrombosis and accelerated arteriosclerosis (3, 7, 198) (**Figure 2**). In addition to the changes in the vessels, fibrosis, thinning, and atrophy of the skin layers were described as pathological findings. Another frequently documented characteristic of CR in VCA is the increased appearance of tertiary lymphoid organs (TLOs) (i.e., highly ordered structures resembling the cellular composition of lymphoid follicles) (22). These TLOs are believed to be local sites of alloantibody production and T cell activation (90). Further, cases of simultaneous targeting of skin plus vessels, and isolated damage to cutaneous or vascular structures, respectively, have been reported (198). Accordingly, skin and vasculature appear to be independent CR targets. It is hypothesized that the skin may be more prone to cellular rejection mechanisms, while vascular structures may be more likely to be the target of humoral immune responses. Although it is widely agreed upon that vasculature and skin are main targets of chronic rejection in VCA, there is little evidence that vascular changes are necessary for chronic changes of the skin component. In a case published by our workgroup, we thoroughly studied the explant of a facial VCA that was removed after ten years following the patient’s death due to unrelated reasons (194). There was no graft vasculopathy despite obvious chronic skin changes (pale appearance of the graft, telangiectasis, epidermal thinning, loss of rete ridges, papillary dermal sclerosis) and numerous acute rejection episodes over the course of follow-up. Repeated (sub-)clinical acute T-cell mediated rejection of the skin part was hypothesized to have induced a profibrotic change over time. Given steady motor function scoring, it seems like underlying musculature was spared of such chronic changes. Thus, we assumed that two phenotypes of chronic rejection may exist, one that involved chronic immune-mediated arteriosclerotic change of the vasculature and one that spares the vasculature and predominantly affects the skin component. While acute VCA rejection is mainly based on the interaction of CD8^+^ effector T cells targeting follicular and epithelial stem cells, T and B cells are likely to represent the primary effectors in chronic rejection (40). In contrast, EC may be considered a primary target during chronic rejection with changes of vasculature. However, there are also chronic pathologic changes (e.g., loss of rete ridges) independent of the vasculature (194). Thus, the cellular landscape of chronic VCA rejection represents an ongoing area of research and remains to be further elucidated.

## 14 The need for cell-specific VCA therapies

Detailed knowledge of cellular crosstalk after VCA surgery is critical to further improve allograft survival, functional long-term results, and patient-reported outcomes (199–201). The current standard immunosuppressive drug regimen based on SOT protocols consists of anti-thymoglobulin/alemtuzumab/rituximab combinations as induction therapy, followed by a high-dose maintenance protocol, most commonly including tacrolimus, MMF, and prednisone (5, 15). Commonly, is recommended to maintain high tacrolimus levels (10–15 ng/ml) during the first three months posttransplant and then tapering it down to 5–10 ng/ml. Prednisone doses are also tapered to be maintained at lower doses (5–15 mg/day) for six to twelve months in the majority of VCA recipients. Based on these general considerations, individual therapy protocols are administered to minimize renal side effects, stabilize glycemic control, and prevent neurotoxicity as well as myointimal hyperproliferation of the vasculature (202–204). Despite extensive immunosuppression, about 85% of VCA recipients experience at least one episode of acute allograft rejection in the first year. This might be due to certain cell types being less sensitive to, or even triggered by, current immunosuppressive protocols. For example, tacrolimus mainly targets T cell subsets by inhibiting the IL-2 activation pathway, whereas Wai et al. found tacrolimus has no effect on NK cell cytotoxicity (205, 206). While corticosteroids have been shown to efficiently hinder T_h_ cell immunity at various stages of the activation cascade and impair cytokine production, as well as effector function, their effects on B cells seem to be more complex (207). Cupps et al. found corticosteroids to have only minor effects on B cell proliferation and even drive immunoglobulin secretion when stimulated with B cell growth factor *in vitro* (208). Interestingly, Cooper et al. have demonstrated that prednisone can indeed decrease NK cell concentration but does so only in a minority of patients (209). In contrast, MMF has been shown to enhance cytotoxicity and chemotaxis in NK cells (210). Thus, more profound knowledge of cell types involved in VCA tolerance and rejection may help to target the respective cellular subsets and reduce medication side effects more specifically. Of note, there has been little change regarding the immunosuppressive drug regimen administered following VCA surgery for the past two decades. For more detailed information, please refer to some elegant work by Kauke-Navarro et al. (5).

The role of each cell types within the context of the respective tissue is important. For example, Lian et al. identified that T_rm_ in skin tissue is a pivotal cell subset in facial VCA rejection (12). Yet, the same cell subset might have different or even opposite functional and phenotypic properties in the mucosa, which has been recently identified as another promising target to modulate the VCA rejection reaction (14, 15, 18, 19). Further, our knowledge on the relevance of passenger immune cells (PICs) in acute and chronic rejection remains limited. PICs refer broadly to all the graft-derived immune cells that are transferred to the host secondary lymphoid tissue and trigger allograft rejection by direct recognition of the alloantigen (211, 212). However, PICs seem to contribute to allograft rejection and tolerance induction at the same time. In rat liver allograft models, irradiation of the allograft prior to transplantation triggered transplant rejection in otherwise tolerant recipients, underscoring the potential tolerance induction role of donor-derived graft-resident PICs (213, 214). Yet, direct recognition of the alloantigen by CD4^+^ T cells was considered to persist at early time points after SOT and was highly correlated with the lifespan of graft DCs (215). As an alternative pathway to the insult of donor endothelium by recipient immune cells, PICs were hypothesized to attack the recipient endothelium that has chimerically populated donor-derived vasculature during chronic transplant rejection (12). In facial allograft rejection, Lian et al. discovered that immune cells spatially associated with vascular, pilosebaceous, and epidermal sites of injury were mainly T_rm_ (please see “Endothelial Cells” for further details) of donor origin (12). This finding represents a novel research perspective as the current dogma assumes that skin allograft rejection is mediated by recipient T cells. The local association of donor T cells with sites of direct cell injury reinforces the hypothesis of more complex cross-talks between immune cell during the rejection process. Overall, a more advanced and biomarker-based approach to the assessment of VCA rejection is still needed.

## 15 Discussion

**Figure 3:**
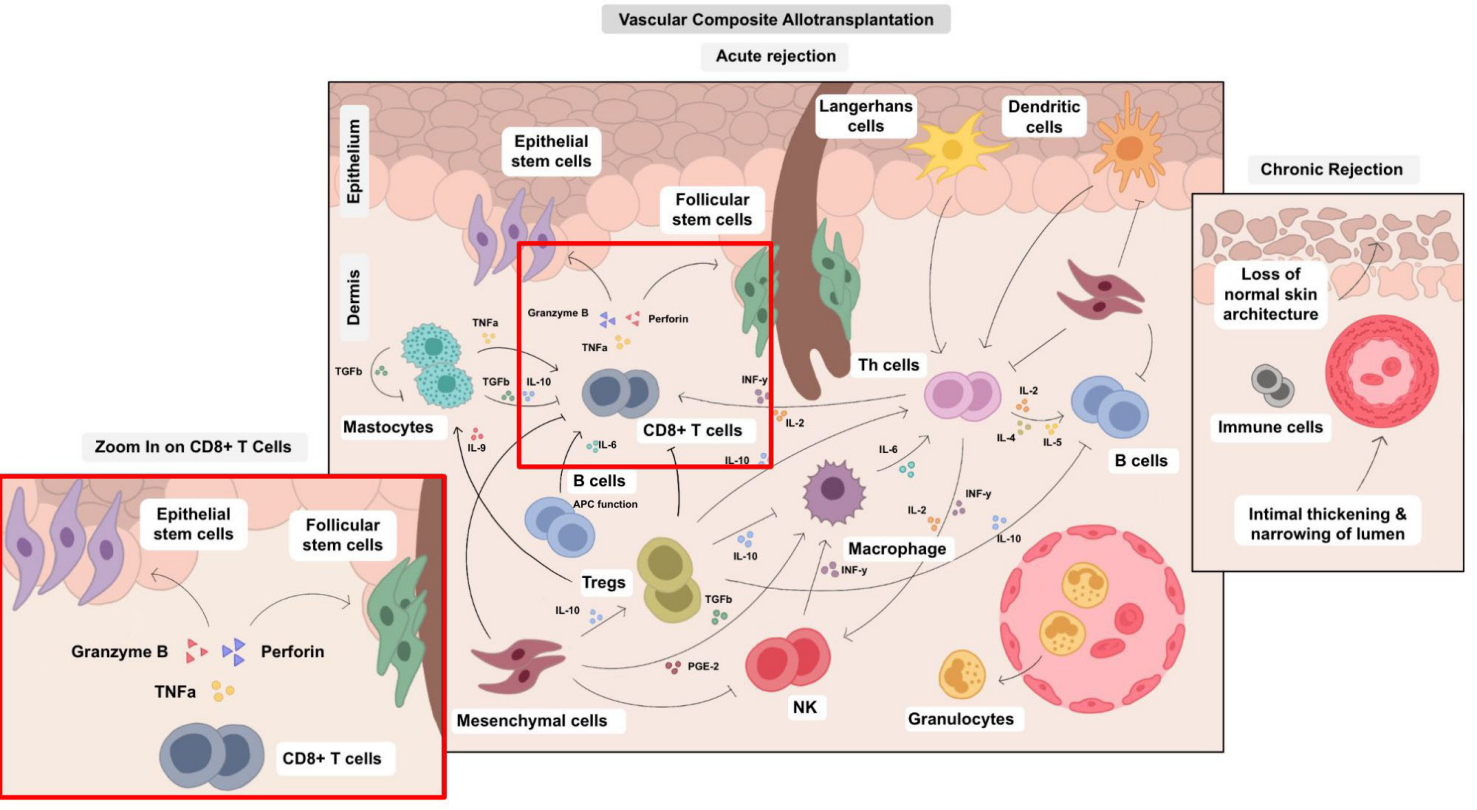
Cellular cross-talks in the field of VCA. The various cell types in VCA interact *via* multiple pathways and their balance determines surgical VCA outcomes. Therefore, an isolated approach impacting single cell types is a challenging, yet potentially high-yield leverage point. Different cell types are involved both in acute and chronic rejection and can trigger various pathological processes, e.g., epidermal thinning and loss of rate ridges, follicular plugging, hyperkeratosis, vascular ectasia subepidermal sclerosis with collagen type I deposition and collagen type I shift into the papillary dermis as published by Krezdorn et al., 2019 (65).

The cell types relevant to VCA are multifunctional and interact through complex pathways (**Table 1** and **Figure 3**). It is important to note that each cell line represents a mere piece of the puzzle, however a clear hierarchy of effector-target cell interactions can be formulated based on current understanding of the pathomechanisms of acute and chronic cellular rejection in VCA. Some cells are more important than others with CD8^+^-T-cells posing as the protagonists, mainly targeting epithelial and follicular stem cells. Researchers and clinicians should be aware of this complex, closely interlinked network. Despite the predominance of T-cells and effective suppression by current immunosuppressive regiments, it is important to note that other cell types fuel the rejection process and further studies are needed to define the exact impact of such secondary effector (and target cells). Rejection of VCAs frequently occurs and several longer-term studies demonstrate chronic allograft changes. This demonstrates that current treatment regimens are not perfect at mitigating the immune response, indicating that other cell types not currently (sufficiently) targeted by the standard triple maintenance regimen, may be more relevant than previously thought. Future studies are needed to better define the cellular network leading to acute and chronic VCA rejections.

**Table 1:** Summary of cell types involved in acute and chronic rejection of vascularized composite allotransplantation (VCA).

Type of Cell	Function
Stem Cells 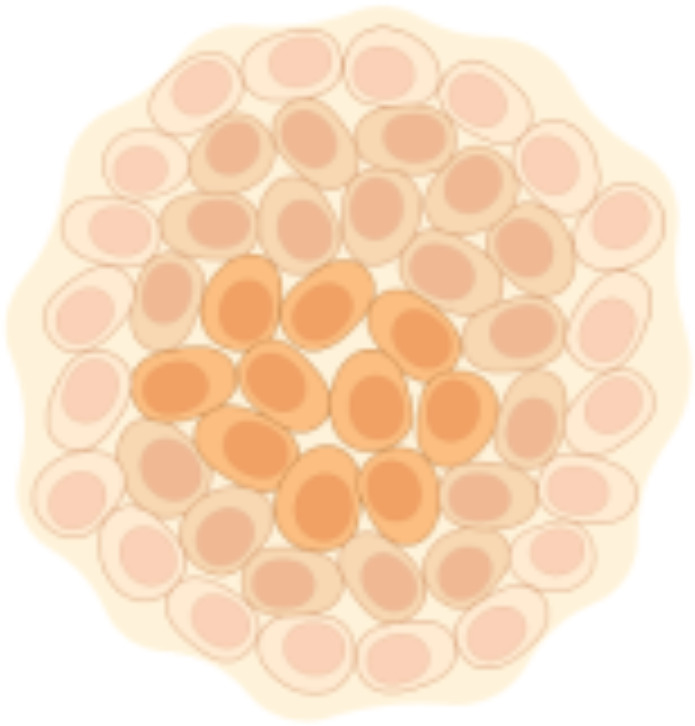	Primary target cells [58]. Important role in skin regeneration, and immunology with MHC class I and II expression increased under pathological conditions [51]. Cross-talks with various immune cell types including ^a^ and macrophages are mediated via different pathways (e.g., JAK- STAT, β-catenin/Wnt, and Jag1-Notch) and mechanisms (e.g., expression of leukocyte differentiation antigens and ALCAM) [61]. Apoptosis of stem cells can be induced by different cell types such as T cells (e.g., via CD106) with proinflammatory cytokines (e.g., TNF-α) priming stem cells towards apoptosis [69].
T Cells 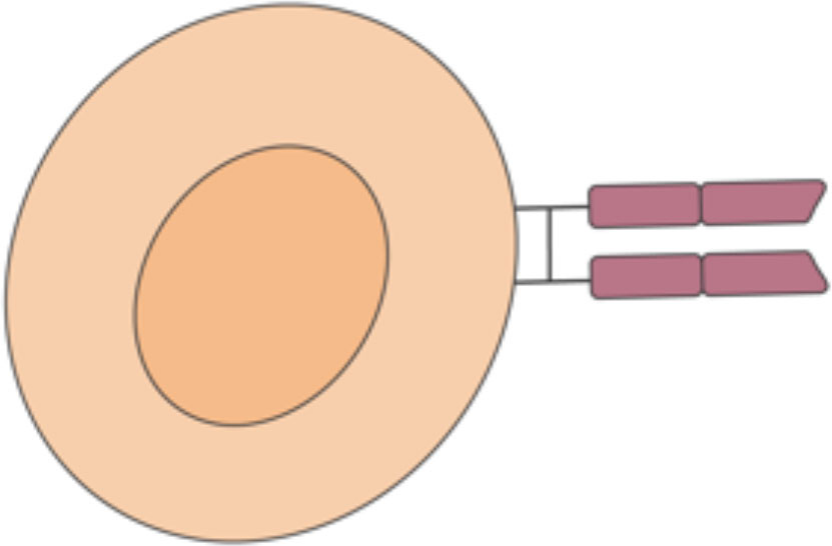	Primary effector cells (especially CD8^+^ T cells) [43]. Recognition of donor-derived processed antigen fragments and/or intact antigens [44]. Involvement of donor- and recipient-derived T cells in the rejection process surrounding epithelial target cells (i.e., satellitosis) and graft vasculature [8]. In higher-grade rejection, T cell activation is based on the upregulation of genes associated with T cell infiltration, co- stimulation, and cytotoxicity [13].
Endothelial Cells (EC) 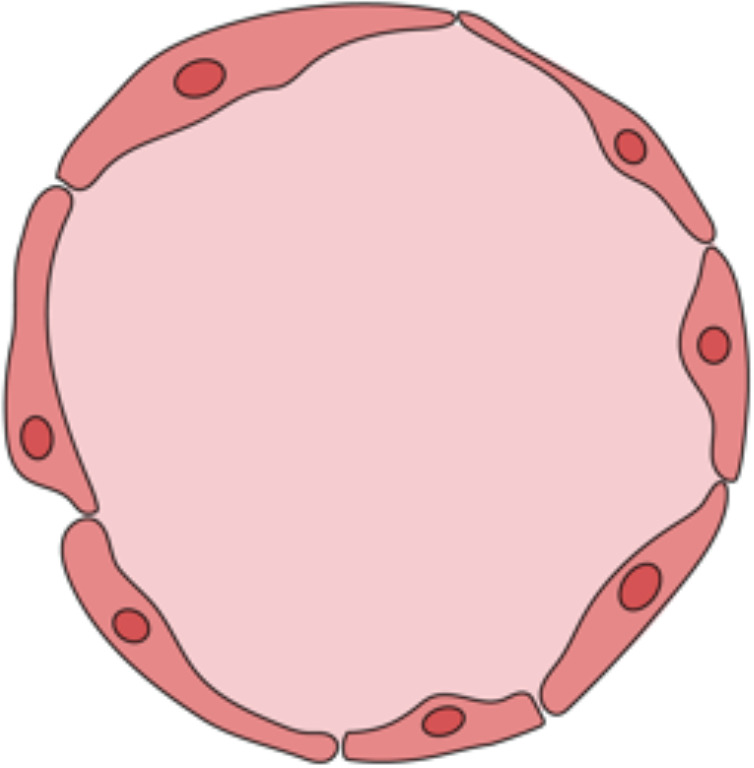	Activation and recruitment of lymphocytes via upregulation of HLA-II and adhesion molecules [12]. Increase of nitric oxide levels driving the dismantling of intraendothelial adherens junctions and resulting in disruption of the endothelial barrier [82]. Reactivation of resting memory T cells [86]. Drivers of fibrosis and thrombosis damaging the VCA vessels [91]. Interaction with lymphoid follicle formation during chronic rejection phase [92].
Antigen Presenting Cells (APC) 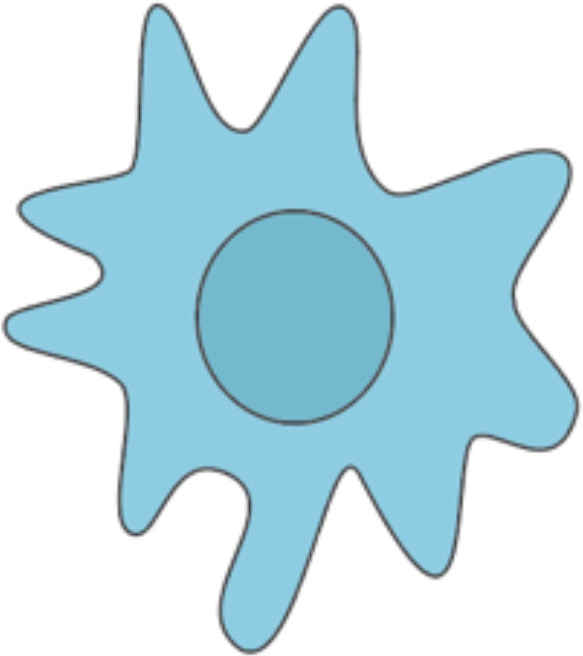	Activation of alloreactive recipient-derived T cells [141]. Dendritic-appearing CD8^+^ T cells as subclass of dendritic resident memory T cells implicated with early-onset VCA rejection [12]. Loss of CD169^+^ tissue-resident macrophages as indicator of VCA rejection [146]. Production of IL-18 and reactive oxygen species driving pro- apoptotic and pro-inflammatory pathways [148].
Natural killer (NK) Cells 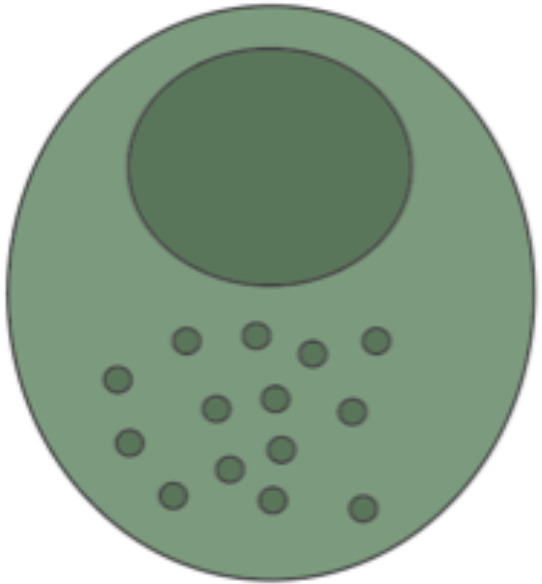	Navigation to the site of inflammation via the interaction of C-X3-C motif receptor-1 and CX3CL-1 expressed by EC [221]. Production of IFN-γ promoting CX3CL-1 expression on EC [221]. Secretion of granzyme B regulating cell apoptosis and increasing pro-apoptotic Bid [109].
Granulocytes as APC- like Cells 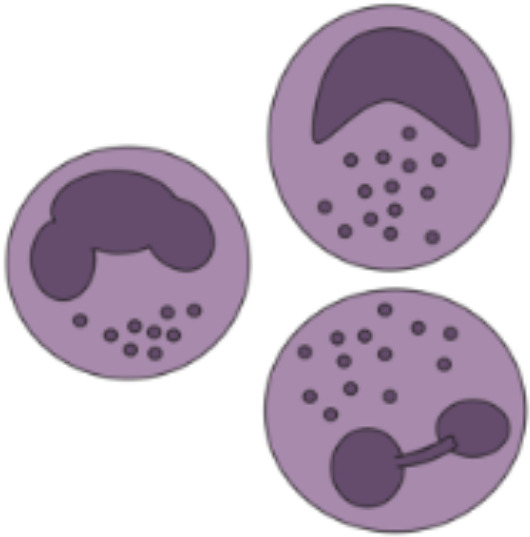	High overall percentage of granulocyte amongst infiltrating leukocytes in inflamed VCA graft regions [106]. Located in the interstitium and toward the epidermis [163]. Correlation of neutrophilic inflammation and severe VCA rejection in animal models [164].
B Cells 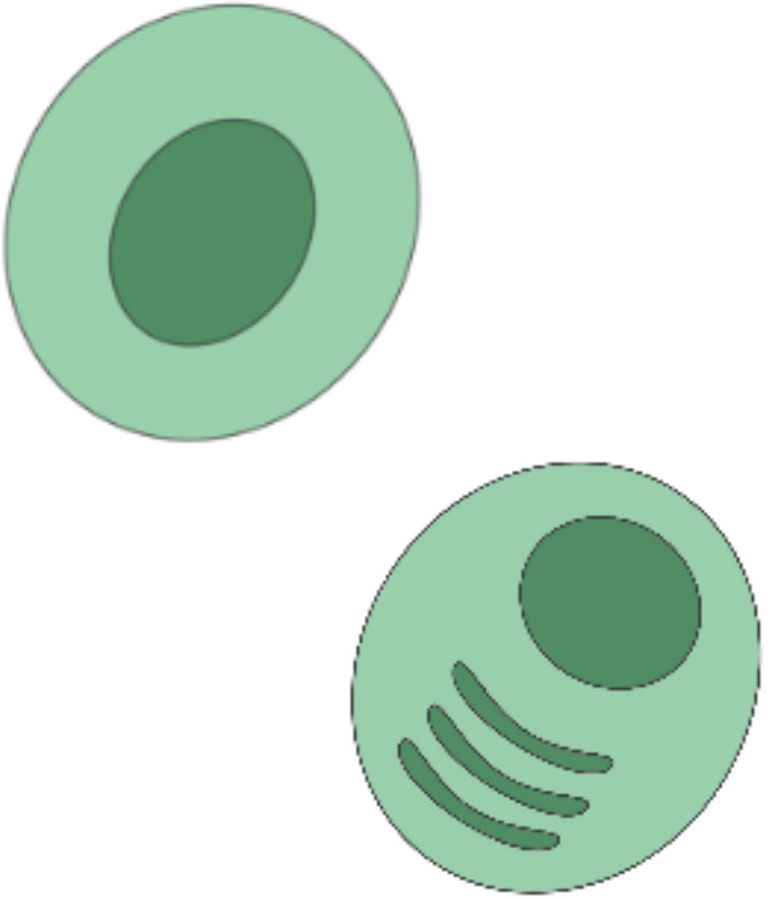	Production of donor-specific antibodies [123]. T_h_17 subset as drivers of B cell proliferation, antibody production, inducible T cell costimulator (ICOS) molecule reinforcing B cell differentiation [131]. Formation of tertiary lymphoid organs (pathological hallmarks of chronic VCA rejection) through CD20^+^ B cells [90]. Acting as professional APC which are recognized by recipient T cells through HLA molecules and simultaneously present donor-derived antigens to recipient T-cells [126].
Passenger Immune Cells 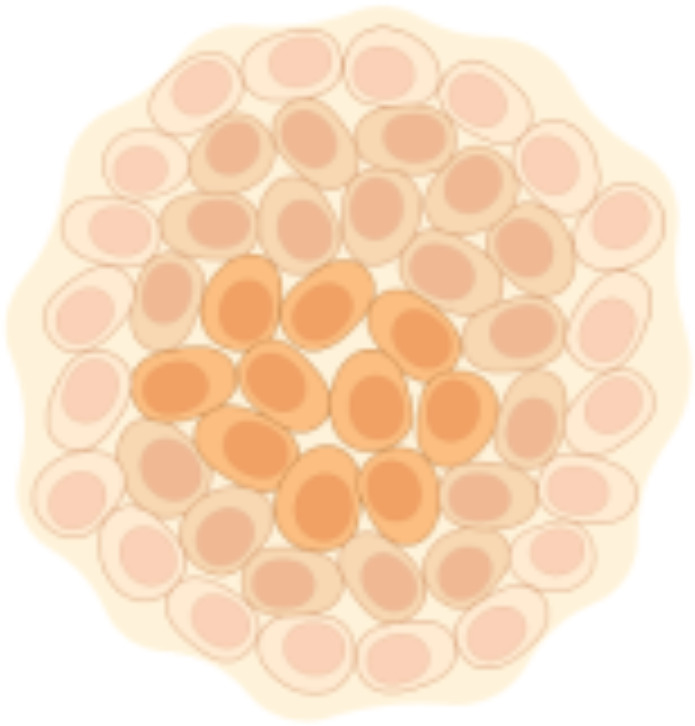	Presumed to have double-edged role as contributor to both allograft rejection and tolerance induction [218]. Tolerance-inducing function in murine SOT model [219]. Hypothesized attack on the recipient endothelium chimerically populated with donor-derived vasculature during chronic rejection [12].
Additional Cell Types: Regulatory T cells (Tregs) Mesenchymal Stem Cells (MSC) Adipose-derived stem Cells (ASC) Langerhans Cells (LC) Mast Cells (MC)	T_regs_ (CD4^+^CD25^+^CD127^-^ T cells) have been shown to prevent skin allograft rejection in murine models and can be equipped with a chimeric antigen receptor (CAR) [171]. MSC have been demonstrated to mitigate acute allograft rejection with augmented microvascular blood flow and oxygenation of the graft ultimately significantly prolonging graft survival [178]. ASC seem to prolong VCA graft survival and exert attenuating effects on T cell proliferation while increasing T_regs_ and B_regs_ counts [180]. LC can mediate immunosuppressive effects by promoting the expansion and activation of skin-resident T_regs_ [189]. MC represent a major source of TNF-α in the skin and thus may promote allograft rejection by inducing proinflammatory

## Author’s own work

The authors state that this manuscript represents their own work. **Figure 1** has already been published by Lian et al., while all the other figures and supplements have been created by the author’s team (12).

## Author contributions

LK: writing and manuscript preparation. SK: writing and manuscript preparation. AP: reviewing and figures. CaL: reviewing and figures. SS: histopathological background. LH: cellular background. VS: reviewing and immunity background. AS: reviewing immunity data. BK: reviewing immunity data. VM: reviewing rejection paragraphs. ChL: reviewing rejection paragraphs. ST: reviewing. GM: reviewing and figures. BP: reviewing and manuscript. MK-N: reviewing and manuscript. All authors contributed to the article and approved the submitted version.

## Supplementary material

Supplementary Figure 1 Flow chart. The key points of this work include the definition of types of rejection in VCA surgery followed by an in-depth description of target and effector cells for acute and chronic rejection reactions.
