# Supplementary figures and images for: Cellular activation pathways and interaction networks in vascularized composite allotransplantation
Source: Front Immunol. 2023 May 17;14:1179355. doi: 10.3389/fimmu.2023.1179355 (PMC10230044; doi:10.3389/fimmu.2023.1179355)

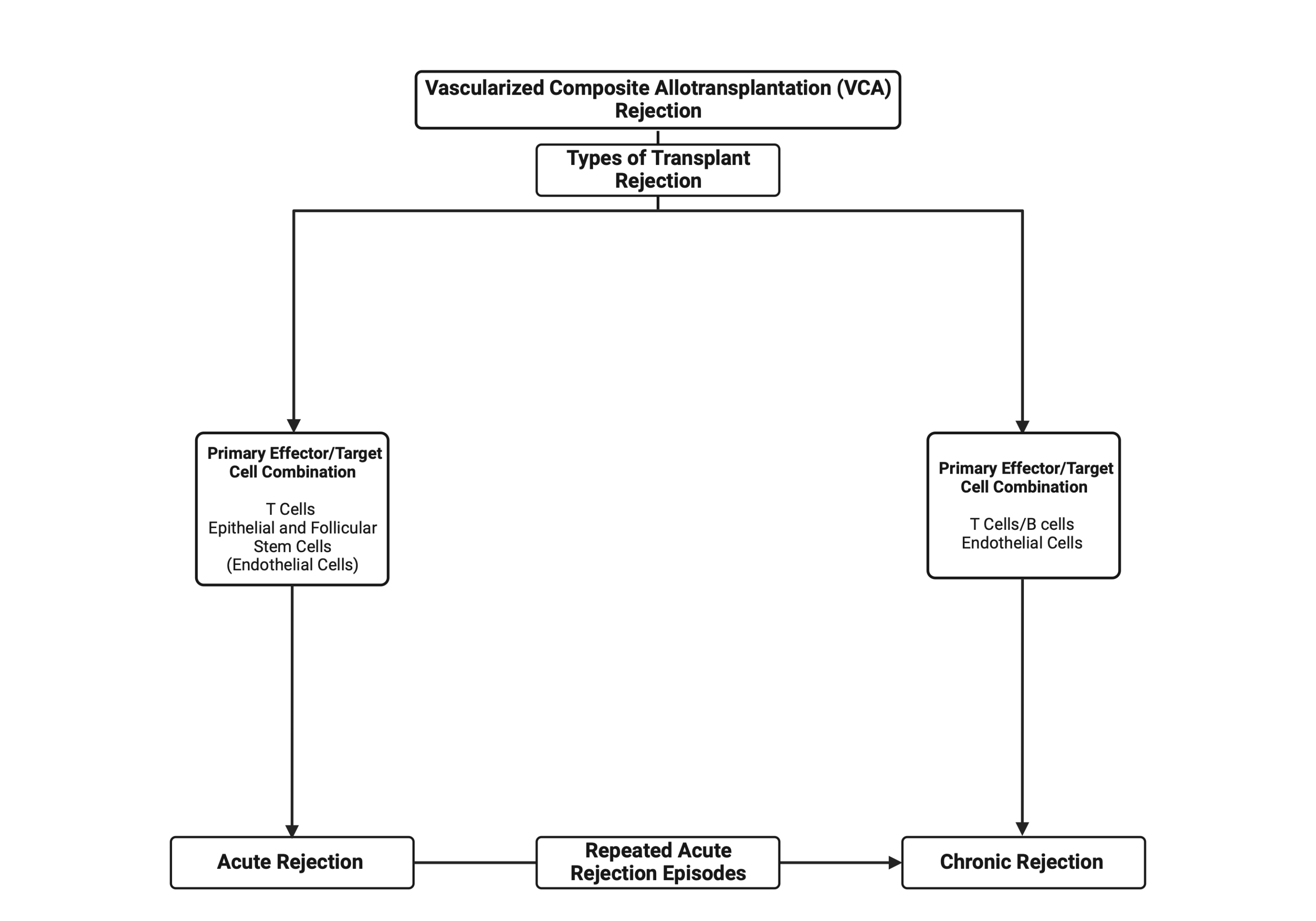

Supplement: Supplementary Figure 1 — Flow chart. The key points of this work include the definition of types of rejection in VCA surgery followed by an in-depth description of target and effector cells for acute and chronic rejection reactions. [file Image_1.jpeg]
